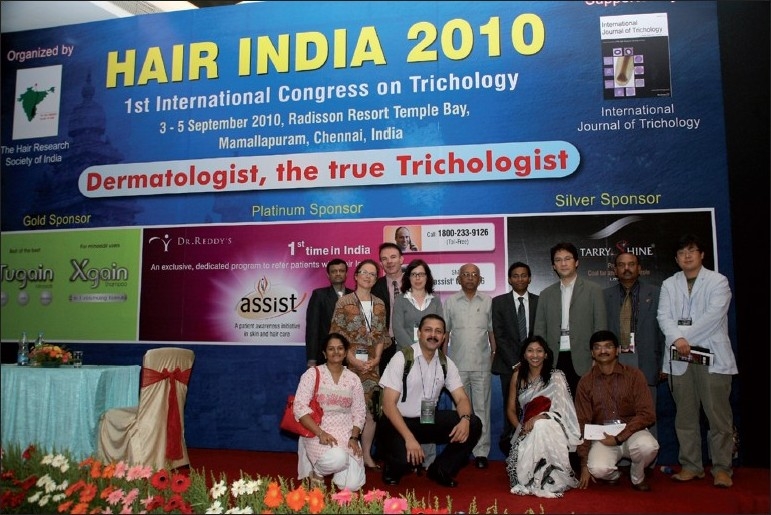# Hair India 2010

**DOI:** 10.4103/0974-7753.77508

**Published:** 2010

**Authors:** Patrick Yesudian

**Affiliations:** President, The Hair Research Society of India, No 10, Ritherdon Avenue, Vepery, Chennai 600 007, India. E-mail: patnirmu@gmail.com

Hair India 2010, the first international conference held by the Hair Research Society of India was organized by the secretary Dr. S Murugusundram in the pristine surroundings of the Shore Temple, washed by the waters of the Bay of Bengal, at the historical seventh century port city, Mamallapuram; a monumental marvel of rock-cut monoliths on the coramandel coast. Nearly 650 delegates and faculty from different parts of India, neighboring countries like Bangladesh, and other hair societies around the world attended the conference. It was unanimously acclaimed to be a great success. We hope to hold the next conference two years hence.

In the inaugural address, I listed the reasons for starting the Hair Research Society, primarily among which were the myths and misconceptions about hair biology and hair diseases among the lay public and our non-dermatological medical colleagues, leading to quackery and unscientific treatment for trichological problems. The lay press was partly responsible for this. Although the opening of a hair dressing salon warranted a half page news item in the popular National dailies, no mention was made about the scientific deliberations at Hair India 2010, where the international faculty discussed the latest advances in hair disorders. The priorities of the news media appeared to be rather skewed.

To begin with, a pre-conference workshop on trichology was conducted for a limited number of 100 aspirants. The participants experienced demonstrations and hands on training on hair diagnostic and therapeutic procedures.

The opening session of the conference started with brief presentations of the anatomy, physical properties, and the chemistry of hair. Prof. Regina Betz then spoke about the new genes that have been identified in the last decade, mutations in which cause different types of congenital hypotrichosis. In this session several case presentations of genotrichoses like the Naxos, Nethertons, and Carvajal syndromes were made, and their pathogenesis discussed.

Prof. Desmond Tobin presented the hair-stress nexus, wherein he pointed out that the hair follicle, similar to the skin can utilize locally produced neuroendocrine hormones to neutralize the noxious stimuli and is an important component of the neuroimmunoendocrine pathway.

Prof. Won Soo Lee presented his new basic and specific (BASP) classification of pattern hair loss and said that it was easier to follow than the previous classification, for this common hair problem.

Prof. Ramon Grimalt’s case presentations of unusual clinical trichological problems drew audience participation.

Prof. Lidia Rudnicka elaborated the importance of the trichoscope, which, if properly wielded by the dermatologists, can act as their magic wand. She said that trichoscopy not only aids in the accurate diagnosis of many hair disorders, but also allows us to assess disease activity and to monitor treatment efficacy.

Prof. Hemangi Jerajani presented a novel method of studying hair diseases using noninvasive ultrasonography. It could also be used for visualizing treatment efficacy.

Dr. S Murugusundram showed the new simplified approach of using a trichogram with a handheld dermascope, which would enable a busy clinical trichologist to quickly arrive at a diagnosis while treating complex hair disorders.

Prof. Rachita Durat emphasized the difficulty of accurately diagnosing female pattern hair loss because of the variable presentations and its multifactorial and polygenic etiology.

There were several articles in psycho-tricho dermatology, which reinstated the influence of mind on hair disorders.

The highlight of the presentations on cicatricial alopecia (CA) was by Prof. Prathima Karnik of the Cicatricial Alopecia Research Foundation (CARF), who gave insights into the pathogenesis of carcinoma (CA) and the role of peroxisome proliferator-activated receptor (PPAR) Gamma-regulated pathways in the etiology of lymphocyte mediated CA.

Besides these, many other facets of Trichology, both medical and surgical, were presented, which held the audience spellbound.

A humorous yet thought-provoking note on hair by Prof. Ganesh Pai and a unique debate on the attractiveness of hair were other notable aspects of the conference.

A new trend was created by subdividing the super speciality of trichology into its branches, namely, Trichomycology, Trichopathology, Tricho Immunology, Pediatric trichology, Cosmetic trichology, Tricho Pharmacology, Tricho Gerontobiology, Nutrition in Trichology, Tricho Lasers, and Neotrichology or Future trichology, with lectures delivered by eminent specialists in their respective fields. The essential technical aspects of clinical trichology such as Tricho Photography was taught and demonstrated.

Most of the sessions had vibrant panel discussions, which involved the audience with enthusiastic academic exchange.

Nine articles of original research were chosen for the award presentations, of which the first prize was awarded to Dr. Punit Saraogi for his study on the ease versus errors of Trichoscan, and the second prize went to Dr. Deepal Deshpande who established newer approaches in treating severe resistant alopecia areata. There were many interesting free communications by young researchers. There were 40 poster presentations. The best poster was on the Chediak Higashi syndrome by Dr. C H Hymavathy.

The hot topic of controversies in trichology with evidence-based trichology and ethical issues were discussed before the grand finale. A quiz on the spectrum of clinical hair disorders was open to the floor.

A potpourri of Indian classical music and dance was presented as a cultural feast relished by the national and International delegates.

As all good things come to an end, Hair India 2010 [Figure 1] also did, with the vote of thanks by our dynamic secretary whom I would like to commend for his sincere and untiring efforts in making this a grand success. I would rather call it a pause and not an end because we have already started preparing to welcome you all for Hair India 2012!

**Figure 1:**